# Novel Antifungal Activity of *Lolium*-Associated *Epichloë* Endophytes

**DOI:** 10.3390/microorganisms8060955

**Published:** 2020-06-24

**Authors:** Krishni Fernando, Priyanka Reddy, Inoka K. Hettiarachchige, German C. Spangenberg, Simone J. Rochfort, Kathryn M. Guthridge

**Affiliations:** 1 Agriculture Victoria, AgriBio, Centre for AgriBioscience, Bundoora, 3083 Victoria, Australia; krishni.fernando@agriculture.vic.gov.au (K.F.); priyanka.reddy@agriculture.vic.gov.au (P.R.); inoka.hettiarachchige@agriculture.vic.gov.au (I.K.H.); german.spangenberg@agriculture.vic.gov.au (G.C.S.); simone.rochfort@agriculture.vic.gov.au (S.J.R.); 2 School of Applied Systems Biology, La Trobe University, Bundoora, 3083 Victoria, Australia

**Keywords:** *Lolium* spp., perennial ryegrass, tall fescue, *Epichloë* spp., endophyte, disease resistance, antifungal activity, metabolome, antifungal metabolites

## Abstract

Asexual *Epichloë* spp. fungal endophytes have been extensively studied for their functional secondary metabolite production. Historically, research mostly focused on understanding toxicity of endophyte-derived compounds on grazing livestock. However, endophyte-derived compounds also provide protection against invertebrate pests, disease, and other environmental stresses, which is important for ensuring yield and persistence of pastures. A preliminary screen of 30 strains using an in vitro dual culture bioassay identified 18 endophyte strains with antifungal activity. The novel strains NEA12, NEA21, and NEA23 were selected for further investigation as they are also known to produce alkaloids associated with protection against insect pests. Antifungal activity of selected endophyte strains was confirmed against three grass pathogens, *Ceratobasidium* sp., *Dreschlera* sp., and *Fusarium* sp., using independent isolates in an in vitro bioassay. NEA21 and NEA23 showed potent activity against *Ceratobasidium* sp. and NEA12 showed moderate inhibition against all three pathogens. Crude extracts from liquid cultures of NEA12 and NEA23 also inhibited growth of the phytopathogens *Ceratobasidium* sp. and *Fusarium* sp. and provided evidence that the compounds of interest are stable, constitutively expressed, and secreted. Comparative analysis of the in vitro and in planta metabolome of NEA12 and NEA23 using LCMS profile data revealed individual metabolites unique to each strain that are present in vitro and in planta. These compounds are the best candidates for the differential bioactivity observed for each strain. Novel endophyte strains show promise for endophyte-mediated control of phytopathogens impacting *Lolium* spp. pasture production and animal welfare.

## 1. Introduction

Asexual *Epichloë* spp. endophytes (previously *Neotyphodium* spp.) provide a competitive advantage to their host plant via production of bioprotective compounds that defend the host plant against disease-causing phytopathogens, herbivory by animals and insects, and abiotic factors such as water and mineral stress [1,2,3,4,5]. *Epichloë* spp. grow in association with agriculturally important pasture grass species including perennial ryegrass (*Lolium perenne*), Italian ryegrass (*Lolium multiforum*), meadow fescue (*Lolium pratense*), and tall fescue (*Lolium arundinaceum*). Endophytes in this genus exhibit host specificity such that the asexual *Epichloë* species are fairly selective to the species of host they colonize; however, some species such as *Fa*TG-3 are able to stably colonize other *Lolium* spp. via artificial inoculation (Table 1) [6].

In the grass-endophyte association, the endophyte produces alkaloids that are beneficial to the growth and persistence of the host grass. Among the alkaloids produced, research has focused on four classes of alkaloids: the indole-diterpenes (lolitrem B and epoxy-janthitrems), ergot alkaloids (ergovaline), 1-aminopyrrolizidines (often referred to as lolines), and the pyrrolopyrazine alkaloid (peramine). Some endophyte-derived alkaloids (i.e., lolines, ergovaline, peramine, epoxy-janthitrems) confer invertebrate resistance to the host plant by deterring feeding or affecting the invertebrate lifecycle [7]. Lolitrem B and ergovaline are the predominant causative agents of ryegrass staggers and fescue toxicosis respectively [8,9,10,11]. Consequently, these major classes of alkaloids have been studied extensively for their toxicity to animals and invertebrates, while knowledge regarding the beneficial properties of other endophyte-derived secondary metabolites, such as epoxy-janthitrems, remains limited [11,12].

Grasses are threatened by many phytopathogenic microorganisms that cause severe damage to the plant, these include crown rust (*Puccinia coronata*), stem end rust (*Puccinia graminis*), grey leaf spot (*Pyricularia grisea*), brown blight and net blotch (*Drechslera* sp.), blind seed disease (*Gloeotinia temulenta*), yellow patch (*Ceratobasidium cereale)*, snow mould (*Microdochium nivalae*), damping off (*Fusarium oxysporum*), bacterial wilt (*Xanthomonas transluscens*), and ryegrass mosaic virus (RgMV) (Table 2) [12,13,14,15]. These diseases lead to reductions in forage quality as well as yield (Table 2).

Fungal phytopathogens in pastures also impact on animal health and wellbeing by producing mycotoxins. For example, *Fusarium* sp. pathogens produce the toxins T-2/HT-2, zearalenone, deoxynivalenol and derivatives that affect food intake and performance in dairy cattle, leading to low milk production and reduced weight gains [41,42]. Some diseases such as rusts reduce the palatability of pasture by altering soluble carbohydrate content, which then affect milk production [43]. Rust diseases also contribute to increased dead plant material in the field which facilitates growth of saprophytic fungi such as *Pithomyces chartarum. P. chartarum* causes facial eczema externally and damages the liver and bile ducts of cattle upon consumption [44]. In addition to the detrimental effects on animal health and production, consuming animal products exposed to mycotoxin contaminated feed may impact human health [45,46].

Most studies into the antifungal bioactivity of *Epichloë* spp. focused on selected strains of sexual *Epichloë* spp., such as *E. typhina* and *E. bromicola,* rather than the symbiotic asexual species utilized in *Lolium* spp. pastures [47,48]. These studies identified the antifungal potential of selected strains using in vitro assays [49,50] and in some cases, isolated compounds that were effective against pathogens in in vitro culture using plate-based assays [5,47,51]. Previously reported fungicidal compounds produced by sexual *Epichloë* spp. that may provide protection from disease include sesquiterpenes, indole-3-acetic acid, chokol A-G, and gamohonoloides [47,48,50,51,52,53].

Early studies indicate that major known alkaloids such as lolines, peramine, and ergot alkaloids do not exhibit antifungal activity against *Colletotrichum graminicola, Limonomyces roseipellis, Ceratobasidium cereale, and Rhizoctonia zeae* in culture [54]. This observation suggests *Epichloë* spp. endophytes produce undiscovered antifungal metabolites in addition to the major alkaloid classes already described [54]. As endophytes play a major role in farming systems, and with the rising interest in utilizing endophytes and endophyte-derived metabolites in crop bio-protection, more bioactive compounds await discovery in asexual *Epichloë* spp. strains that will benefit pastoral agriculture [4,12].

The aim of this study is to investigate the antifungal activity of novel asexual *Epichloë* spp. strains using in vitro bioassays coupled with metabolite profile comparisons. Following preliminary investigations for bioactivity using an in vitro dual culture bioassay, the novel strains NEA12 (*Lp*TG-3) and NEA23 (*Fa*TG-3) were investigated in further detail. NEA12 and NEA23 exhibit differential bioactivity toward the examined pathogens, indicating that the compounds responsible for bioactivity are likely different for each strain. Crude extracts from liquid cultures of NEA12 and NEA23 also differentially inhibit the growth of *Ceratobasidium* sp. and *Fusarium* sp. and provide evidence that the antifungal compounds of interest are both constitutively expressed and secreted. Finally, a comparative analysis of the in vitro and *in planta* metabolomes of NEA12 and NEA23 using LCMS profile data revealed individual metabolites unique to each strain. Uniquely produced metabolites are the best candidates for the differential bioactivity observed for each strain. These novel endophyte strains show promise for use in biocontrol of diseases caused by common grass pathogens.

## 2. Materials and Methods

### 2.1. Plant Material

All plant material was obtained from a glasshouse maintained (natural day lengths and a mean temperature of 22 °C) collection at Agriculture Victoria, Bundoora, Victoria, Australia [55].

### 2.2. Pathogens

All pathogens (Table 3) were obtained from the National Collection of Fungi, Bundoora Herbarium, Victoria. Pathogens were stored as solid cultures of potato dextrose agar (PDA) (Sigma-Aldrich, Castle Hill, NSW, Australia) at 22 °C in the dark, and sub-cultured every two months to maintain stocks.

The genus of the pathogens used in this study was confirmed by ITS sequence analysis. The entire region of nuclear ribosomal DNA which comprises both internal transcribed spacers ITS1, ITS2, and the 5.8*S* subunit was PCR-amplified using primers ITS5 and ITS4 [56]. PCR products were purified and sent to Macrogen for sequencing. Sequences were visualized, corrected, and consensus sequences were created using BioEdit Sequence Alignment Editor v. 7.2.5 (2013) [57]. Consensus sequences were used in BLASTN analysis to identify matches to reference specimens in the NCBI database where minimum of 90% query cover having minimum of 99% similarity in sequence [58].

### 2.3. Dual Culture Assay to Identify Epichloë spp. Endophyte Strains with Antifungal Activity (Preliminary Screen)

*Epichloë* spp. strains were isolated and maintained by Agriculture Victoria. Cultures were stored as solid cultures at 22 °C in the dark, and sub-cultured every two months to maintain stocks.

*Epichloë* spp. strains were cultured on PDA plates and grown for three weeks at 22 °C in the dark (temperature and humidity cabinet, Thermoline Scientific, NSW, Australia). Cultures of pathogen were grown for 7 days at room temperature; 25 °C in the dark [59,60] for use in the in vitro bioassay (Table 4). Two small plugs of pathogenic fungal mycelia (5 × 5 mm) were placed either side of the central *Epichloë* spp. culture. Plates were incubated at 22 °C in the dark and observations were taken when a clear inhibition zone was visible [49,61,62]. Inhibition of pathogen growth was qualitatively compared as strong, moderate, and weak to assess the antifungal activity.

### 2.4. Isolation of Selected Bioactive Epichloë spp. Fungi from Perennial Ryegrass (Lolium perenne)

Fresh isolates of selected endophyte strains were used to confirm bioactivity. Plant material for endophyte isolation was obtained as described above. Presence and identity of endophyte was confirmed by extraction of DNA using Qiagen MagAttract DNA (Qiagen, Hilden, Germany) and SNP-based diagnostic testing, KASP analysis (Kompetitive Allele Specific PCR) (KASP™, LGC Genomics, Teddington, UK) in Bio-Rad T100 thermal cycler (T100™, Bio-Rad Laboratories, Inc., CA, USA). Results were analyzed using BioRad CFX Manager software (CFX Manager™ Software, v. 3.1, © 2013 Bio-Rad Laboratories, Inc., CA, USA) for allelic discrimination.

Selected endophyte strains (Table 4) were isolated using methods described in Ekanayake et al. [6]. Tiller cuts were thoroughly cleaned with tap water, surface sterilized (80% ethanol for 1–2 min, followed by 5% NaOHCl for 10–15 min), and dried on filter paper. Inner sheaths were separated and placed downwards on PDA supplemented with 250 mg/L cefotaxime (Sigma-Aldrich, Castle Hill, NSW, Australia). Cultures were then sealed and incubated at 22 °C for 4 weeks. Endophyte hyphae growing out of plant tissues were transferred to fresh PDA. Strain identity of isolated endophytes was confirmed by extracting DNA using a Chelex 100 method (Chelex^®^ 100, Sigma-Aldrich, Castle Hill, NSW, Australia), followed by SNP-based diagnostic testing described above. The isolated strains were stored as solid cultures at 22 °C in the dark and sub-cultured every two months [63].

### 2.5. Dual Culture Assay to Confirm Endophyte Antifungal Activity

The dual culture assay was performed as described above. Five-week-old subcultures of endophyte strains and 7 day old freshly sub-cultured pathogen cultures were used for the bioassay. Two controls were used; (1) pathogen cultured alone without the endophyte, (2) endophyte cultured alone without the pathogen. All bioassay treatments were prepared in replicate (*n* = 5). Plates were incubated at 22 °C and observations were taken daily for eight days. Growth of the pathogenic fungi was observed and photographed over eight days and compared to the control [61]. Measurements of pathogen growth (cm^2^) were analyzed using ImageJ 1x (NIH, Bethesda, MA., USA) [64]. Image data of fifth day of observations was used for measurement of pathogen growth. One-way-ANOVA was performed using Minitab^®^ 19 Statistical Software (Minitab, LLC, State College, PA, USA). Tukey comparison tables at 99% confidence level were used to determine significant differences of antifungal activity. The assay was duplicated at a different time point, with three replicates, to confirm bioactivity.

### 2.6. Crude Endophyte Extract Assay for Antifungal Activity of Selected Endophyte Strains against Pathogenic Fungi

#### 2.6.1. Preparation of Liquid Cultures

Isolated endophyte strains with confirmed identity were grown in PDB (potato dextrose broth) (Sigma-Aldrich, Castle Hill, NSW, Australia) for metabolite extraction. A sterile scalpel blade was used to cut a small section (1 × 1 cm) of the endophyte periphery on a PDA plate and placed into a sterile 1.7 mL Eppendorf tube containing 500 µL PDB. A sterile plastic pestle was used to gently grind the mycelia and agar. Another 500 µL PDB medium was added to the Eppendorf tube containing the ground endophyte. The ground endophytes were distributed in 500 mL of PDB in 250 µL aliquots. Culture vessels were incubated in the dark at 22 °C on a shaker at 150 rpm (Ratek OM11, Adelaide, Australia) for period of 14 days. Culturing was replicated to collect a total of 10 L.

#### 2.6.2. Extraction

The 10 L PDB culture (1 L × 10 batches) was centrifuged at 3836× *g* for 15 min in 4 °C (Avanti™ J-25I, Beckmann Coulter Centrifuge, Beckman Coulter Life Sciences, IN, USA) and the cell pellet was removed from the media supernatant to obtain two sample types to extract; (1) media supernatant (2) mycelial pellets. The frozen and lyophilized, (Freeze dryer ALPHA, Christ, Germany) (Table 5) media supernatant was weighed and extracted using methanol: H_2_O (*v*/*v*, 4:1, 1:3 *w*/*v*). For extraction media supernatant was vortexed (5 min) (Ratek multi tube vortex mixer, MTV1, Boronia, Victoria, Australia) and sonicated (10 min) (SoniClean, 250TD, Thebarton, South Australia, Australia) with the solvent. Separately, the mycelial pellet was weighed (Table 5) into a falcon tube and extracted (methanol: H_2_O, 4:1 *v*/*v*, 1:3 *w*/*v*) using the same method. The extracts were centrifuged (10 min at 5100 rpm in 22 °C) (Sigma 4-16KS, Sigma Laborzentrifugen GmbH, Germany) and the supernatant was collected. A 60 µL aliquot of the cell pellet and media supernatant extracts were transferred into HPLC vials ready for LCMS analysis. In total four extracts were collected from NEA12 and NEA23 liquid PDB cultures; 80% methanol extract of 2-week-old NEA23 PDB culture supernatant (NEA23 MS); 80% methanol extract of 2-week-old NEA23 PDB culture mycelial pellet (NEA23 MP); 80% methanol extract of 2-week-old NEA12 PDB culture supernatant (NEA12 MS); 80% methanol extract of 2-week-old NEA12 PDB culture mycelial pellet (NEA12 MP). All extracts were dried down in a rotary evaporator (Laborota 4000 efficient, © Heidolph Instruments GMBH & Co Kg, Schwabach, Germany) at 39 °C and resuspended in methanol: H_2_O (*v*/*v*, 4:1, 1:3 *w*/*v*) to 1 g/mL concentration.

#### 2.6.3. Agar Well Diffusion Assay for Crude Extracts

The bioassays were conducted on PDA plates that had four equidistant wells (4 mm diameter) made with a cork borer. The wells were sealed using 5 µL of PDA media. A small plug of pathogenic fungal mycelia (5 × 5 mm) was transferred on to the center of the four wells on the PDA media plate and each agar well was filled with 40 µL of extract. The bioassay plates were prepared in replicates (*n* = 5). Plates were incubated at 22 °C for eight days and observations were taken daily from day three. Two negative controls and one positive control were prepared. The negative controls were pathogen cultured in the same way, but agar wells were filled with either 40 µL of sterile distilled water or 4:1, *v*/*v* methanol: H_2_O. Positive controls were prepared by filling the agar wells with 40 µL carbendazim (1 mg/mL) (97%), (Sigma-Aldrich, Castle Hill, NSW, Australia,), an antifungal compound [65,66]. The growth of pathogenic fungi was observed daily for up to 8 days. This assay was duplicated at a different time point to verify the bioactivity using the same extracts.

Measurements of pathogen growth were analyzed using ImageJ 1x software [64] and expressed as area (cm^2^). Pathogen growth measurements were obtained using image data of well diffusion assays against *Ceratobasidium* sp. and *Fusarium* sp. One-way-ANOVA statistical analysis was performed using Minitab^®^ 19 Statistical Software and generated Tukey comparison tables at 99% confidence level to express the significant differences among of the activity among endophyte strains.

### 2.7. Preparation of in Planta Extracts

Perennial ryegrass plants infected with NEA12 and NEA23 were maintained in a glass house at Agriculture Victoria, Bundoora. Tillers from mature plants were harvested into collection tubes and freeze dried for 48 h before grinding (28 Hz for 2 min, Geno Grinder® 2010, Spex Sample Prep, Metuchen, NJ, USA). The freeze dried, ground plant material (20 mg ± 0.2 mg) was extracted twice with methanol: water (80:20, *v*:*v*, 1 mL). The extracts were combined, dried, and reconstituted in methanol: water (80:20, *v*:*v*, 200 µL) [67].

### 2.8. LCMS/MS/MS Data Acquisition and Analysis

All extracts were analyzed on a Vanquish ultra-high performance liquid chromatography (UHPLC) system (Thermo Fisher Scientific, Bremen, Germany) with a binary pump, autosampler and temperature-controlled column compartment coupled with a Thermo Scientific LTQ Orbitrap Velos Pro ion trap MS system (Thermo Fisher Scientific, Waltham, MA, USA; Thermo Fisher Scientific, Bremen, Germany). Extracts were separated using a Thermoscientific Hypersil Gold 1.9 µm, 150 × 2.1 mm column (Thermo Fisher Scientific, Waltham, MA, USA). Chromatographic separation was performed by gradient elution using water with 0.1% formic acid (Sigma-Aldrich CHROMASOLV^®^, Castle Hill, NSW, Australia, HPLC grade) as Solvent A and acetonitrile with 0.1% formic acid (Sigma-Aldrich CHROMASOLV^®^, Castle Hill, NSW, Australia, HPLC grade ≥99.9%) as Solvent B at a flow rate of 0.3 mL/min. Initial conditions were 98% A which was then progressed to linear gradient to 100% B over 11 min, and this was maintained for 4 min before returning to the initial gradient conditions. Injection volume was 3 µL [11].

The MS detector was operated in FT positive mode using full-scan with a mass range of *m/z* 110–2000. MS/MS/MS (MS^3^) and mass resolution was set as 60,000, 30,000, and 15,000 in first, second, and third scan event respectively. The ESI drying gas (N_2_) was set at a flow rate of 7 L/min at 350 °C and nebulizer gas (N2) pressure was set at 45 psig. Capillary, fragmentor, and skimmer voltage was set at 3500 V, 175 V, and 65 V, respectively. Prior to data acquisition, the system was calibrated with Pierce^®^ LTQ Velos ESI Positive and Negative Ion Calibration Solution (Thermo Fisher Scientific™). Mass spectrometry data were acquired using Thermo Xcalibur v. 2.1 (Thermo Fischer Scientific Inc., Waltham, MA, USA) and data were analyzed using Thermo Xcalibur Qual Browser v. 2.1. Acquired data were analyzed using Refiner MS and Analyst (Genedata, Basel, Switzerland) [11,67]. Using Genedata Analyst all data were filtered to omit average signal less than 0.1, and Venn diagrams were created to compare metabolites from endophytes grown in liquid culture (in vitro) and symbiota (in planta) extracts.

## 3. Results

### 3.1. Antifungal Activity of Epichloë spp. Endophytes

A preliminary screen, using an in vitro bioassay, of 30 strains representing the diversity of asexual *Epichloë* species found in association with *Lolium* spp. identified strains with moderate to strong antifungal activity against the three phytopathogens examined (Table S1). Of those strains NEA12 (*Lp*TG-3), NEA21, and NEA23 (*Fa*TG-3 strains) were selected for further investigation as they represent novel strains with favorable known alkaloid profiles and form stable associations with perennial ryegrass (Table 4). These three endophytes are of high value since they do not produce toxins detrimental to animal welfare but retain insect control properties. In addition to those strains, SE, a commonly utilized endophyte in perennial ryegrass pastures was selected as a representative strain of *Lp*TG-1 because of the bioactivity exhibited.

**Figure 1:**
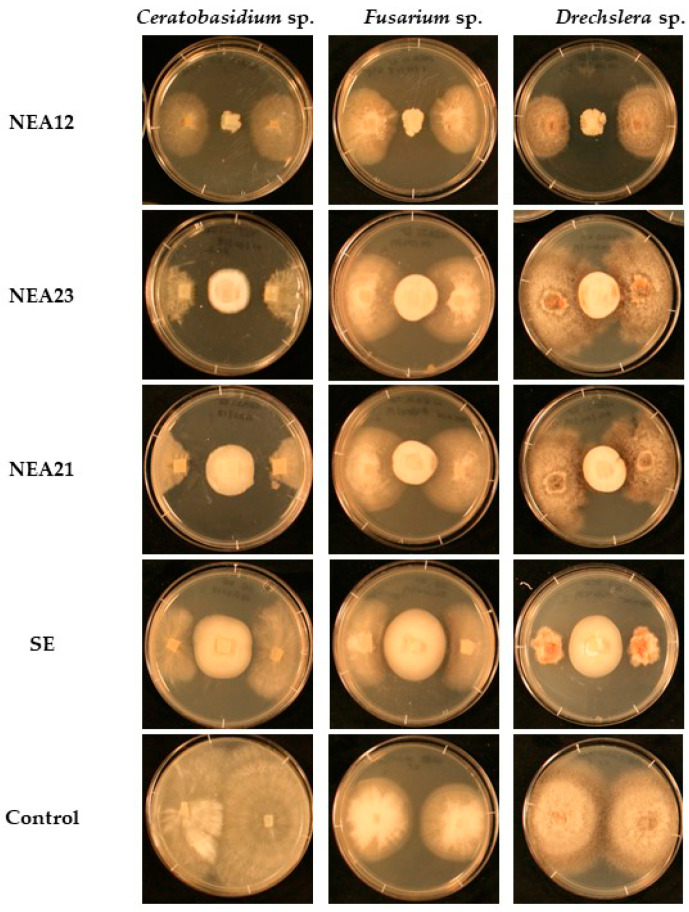
Dual culture assay for growth inhibition of pathogens *Ceratobasidium* sp., *Fusarium* sp*.,* and *Drechslera* sp. by four *Epichloë* spp. strains (in rows) NEA12 (*n =* 5), NEA23 (*n =* 5), NEA21 (*n =* 5), SE (*n =* 5) and negative control (pathogen alone) (*n =* 5) observed on day five of the bioassay. The images are of a typical representative of the five replicates. Refer to Figures S1–S3 for the temporal profile.

**Figure 2:**
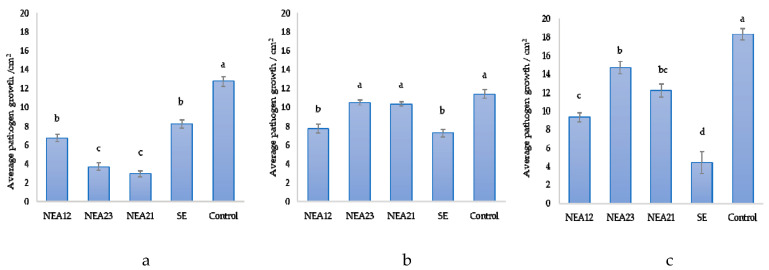
The *Epichloë* spp. endophyte strains NEA12, NEA23, NEA21, and SE show inhibitory activity against (**a**) *Ceratobasidium* sp., (**b**) *Fusarium* sp., and (**c**) *Drechslera* sp. The control in each graph is the pathogen alone. Image analysis was used to measure growth area (cm^2^) of the pathogen on day 5 of the dual culture assays. All data are mean ± standard error, *n* = 5. Means that do not share a letter are significantly different. Significance was determined by one-way ANOVA and Tukey post-hoc test for pairwise comparison; *p* < 0.01 indicates significant inhibition difference between assay results.

Antifungal activity of the four selected strains (NEA12, NEA21, NEA23, and SE) was confirmed using freshly isolated and genetically confirmed endophyte strains (Figure 1, Figure 2 and Figures S1–S3). Identification of the pathogen cultures was also confirmed by ITS sequence analysis, before use.

Visual examination indicated that NEA21 and NEA23 showed strong inhibitory activity against *Ceratobasidium* sp. Inhibition was observed as reduced growth area and density, as well as growth directed away from the endophyte. NEA12 exhibited moderate inhibitory activity against *Ceratobasidium* sp. compared to NEA21 and NEA23. In this case inhibition was observed as reduced growth area and density. For SE, inhibition was observed as a moderate reduction in growth area. One-way ANOVA of pathogen growth area after five days revealed that NEA12 (*p* < 0.001), NEA21 (*p* < 0.001), NEA23 (*p* < 0.001), and SE (*p* < 0.001) exhibited significant pathogen inhibition compared to *Ceratobasidium* sp. grown alone.

Inhibition of *Fusarium* sp. growth was more pronounced in the presence of NEA12 compared to NEA21 and NEA23. In the case of NEA12, visual examination of *Fusarium* sp. growth reveals inhibition as reduced growth area and density, as well growth directed away from the endophyte. Moderate inhibition of *Fusarium* sp. by NEA12 (*p* < 0.001) was significant compared to the control. Statistically, the bioactivity of NEA21 (*p* = 0.125) and NEA23 (*p* = 0.220), as measured by growth area of the pathogen were not significantly different to control samples. However, inhibition is evident by visual examination as reduced density as well growth directed away from the endophyte. The bioactivity of SE as measured by growth area of the pathogen was significantly different to control samples (*p* < 0.001). In this case visual examination also shows inhibition as reduced density and growth directed away from the endophyte.

Visually, inhibitory activity against *Drechslera* sp. by SE was stronger than NEA12, which was in turn stronger than NEA21 and NEA23. The bioactivity of SE as measured by growth area of the pathogen was significantly different from control samples (*p* < 0.001). In this case visual examination also showed inhibition as reduced density, growth directed away from the endophyte, as well as an observable colouration of the pathogen. In the case of NEA12, visual examination of *Drechslera* sp. growth reveals inhibition as reduced growth area and density, as well as growth directed away from the endophyte. One-way ANOVA confirms significant strong activity against *Drechslera* sp. by NEA12 (*p* < 0.001). *Drechslera* sp. growth inhibition by NEA21 and NEA23 was indicated by growth area and density reduction, growth direction of the pathogen and orange pigmentation of the pathogen. Though the activity of NEA21 (*p* < 0.001) and NEA23 (*p* = 0.008, *p* < 0.01) was relatively weak, it was significant compared to the control.

Dual culture assays for each endophyte strain and each of the three pathogens were duplicated at a different time point and consistent results were observed (Figure S4).

Both the preliminary screen and the duplicated confirmation screen using independent isolates showed that *Epichloë* spp. endophyte strains (NEA12, NEA21, NEA23, and SE) consistently displayed antifungal bioactivity, inhibiting the growth of selected pathogens. NEA12 and NEA23 were selected, as representatives of *Lp*TG-3 and *Fa*TG-3, for further investigation of antifungal metabolite production. NEA23 was randomly selected from both *Fa*TG-3 strains as similar phenotypes were observed in the dual culture assays.

### 3.2. Antifungal Activity of Epichloë spp. Endophyte Crude Extracts against Pathogenic Fungi

The antifungal activity observed may be due to secretion of one or more compounds by the *Epichloë* spp. strains examined. Thus, antifungal activity of aqueous methanolic extracts from the media supernatant (MS) and mycelial pellet (MP) of two endophyte strains grown in liquid culture, NEA12 and NEA23, was tested using agar well diffusion assays. In this experiment, the pathogens *Ceratobasidium* sp. and *Fusarium* sp. were selected; growth of *Ceratobasidium* sp. was consistently inhibited by the endophyte strains examined in the in vitro bioassay, while *Fusarium* sp. responded differently to the presence of NEA12 and NEA23.

**Figure 3:**
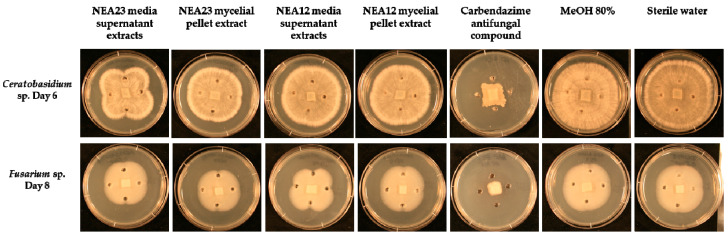
Agar-well diffusion assay for pathogens *Ceratobasidium* sp. (top panel) and *Fusarium* sp. (bottom panel) in the presence of (from left to right): NEA23 MS (*n* = 5); NEA23 MP (*n* = 5); NEA12 MS (*n* = 5); NEA12 MP (*n* = 5); antifungal compound carbendazim (1 mg/mL) (*n* = 5); 80% methanol (*n* = 5); and water (*n* = 5). The images are of a typical representative of the five replicates. Refer to Figures S5 and S6 for full time course.

**Figure 4:**
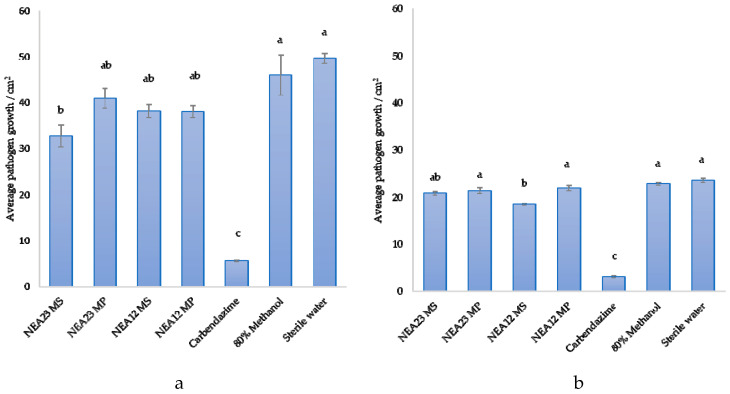
Pathogen growth inhibition by crude extracts from *Epichloë* spp. endophyte strains NEA12 and NEA23. From left to right: NEA23 MS, NEA23 MP, NEA12 MS, NEA12 MP, carbendazime (1 mg/mL), 80% methanol, and water against (**a**) *Ceratobasidium* sp; (**b**) *Fusarium* sp. Image analysis measured growth area (cm^2^) of the pathogen in the agar well diffusion assay. All data are mean ± standard error, *n* = 5. Means that do not share a letter are significantly different. Significance was determined by one-way ANOVA and Tukey post-hoc test for pairwise comparison; *p* < 0.01 indicates significant inhibition.

Extracts of NEA23 MS showed prominent inhibitory activity toward growth of *Ceratobasidium* sp. compared to NEA23 MP, NEA12 MS, and NEA12 MP and the negative controls (Figure 3 and Figures S5 and S6). The distinct x-shaped growth pattern of the pathogen around the agar wells containing crude extracts indicates inhibition. Pathogen growth area, derived from image analysis, was used as a measurable indication of antifungal activity (Figure 4). One-way ANOVA analysis confirmed that pathogen growth inhibition by NEA23 MS was significant (*p* = 0.004, *p* < 0.01) compared to the 80% methanol only control (Figure 4a). NEA23 MP (*p* = 0.680), NEA12 MS (*p* = 0.200), and NEA12 MP (*p* = 0.118) extracts did not significantly reduce growth of *Ceratobasidium* sp.

Extracts of NEA12 MS moderately inhibited the growth of *Fusarium* sp. (Figure 3) showing a reduction in pathogen growth area. The moderate inhibitory activity shown by NEA12 MS against *Fusarium* sp. was significant (*p* < 0.001) compared to the 80% methanol control. NEA12 MP (*p =* 0.862), NEA23 MS (*p* = 0.117), and NEA23 MP (*p* = 0.449) extracts did not significantly reduce growth of *Fusarium* sp. (Figure 4b).

Mycelial pellet extracts from NEA12 and NEA23 did not show any activity against the pathogens examined, indicating that potential antifungal metabolites produced by the endophytes are secreted to the medium and not reserved in large quantities in endophyte cells.

Agar well diffusion assays for each endophyte strain and each of the two pathogens were duplicated at a different time point and consistent results were observed (Figure S7).

The inhibitory activity displayed by NEA12 MS and NEA23 MS are consistent with the dual culture assay results, where NEA23 exhibited stronger bioactivity against *Ceratobasidium* sp. compared to NEA12; and NEA12 inhibited the growth of *Fusarium* sp. while NEA23 did not. Differential inhibitory activity of extracts suggests variation in metabolite composition.

### 3.3. Metabolite Distribution among NEA23 and NEA12 In Vitro and in Planta

In vitro bioassays are of value in determining the bioactivity of isolated and purified endophyte strains, and their extracts, in culture. However, to be of value as a bioprotectant the metabolites must also be produced in planta. LCMS/MS/MS analysis was performed on fungal culture extracts from the media supernatant (NEA12 MS and NEA23 MS) as well as extracts of the corresponding symbiota (NEA12 PE (plant extract) and NEA23 PE) to further investigate the endophyte secretome of the two bioactive strains, NEA12 and NEA23.

Previously described alkaloids known to be unique to each strain were detected in the LCMS analysis. For example, epoxy janthitrem Ⅰ was found in NEA12 in vitro and in planta (Figure S8). Peramine was identified in vitro and in in planta in the NEA23 samples (Figure S9).

**Figure 5:**
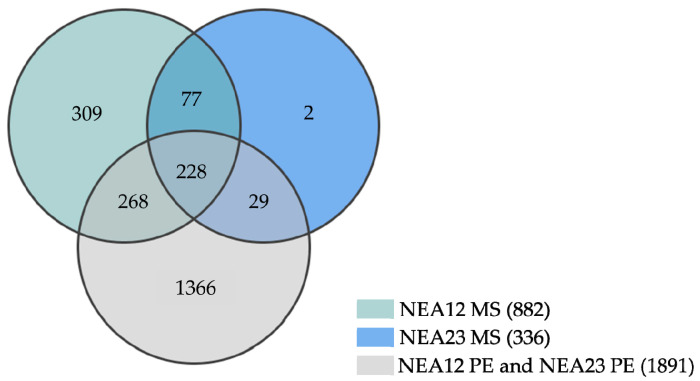
Venn diagram showing the metabolite distribution in NEA12 and NEA23 in planta and in vitro; NEA12 MS (*n* = 2); NEA23 MS (*n* = 2); combined symbiota data from NEA12 PE (*n* = 3) and NEA23 PE (*n* = 3).

A total of 2279 plant-derived and endophyte-derived metabolites were detected in NEA12 MS, NEA23 MS and the combined symbiota data of NEA23 PE and NEA12 PE (Figure 5). Sixty percent of metabolites (1366) were predicted to be plant-derived and 913 endophyte-derived or media constituents based on their presence/absence profiles in the samples studied.

A total of 525 (23%) metabolites were predicted to be endophyte-derived compounds present in both symbiota and in vitro extracts. Of these, 228 (10% of the total) were common to both NEA12 and NEA23, while 268 and 29 were unique to NEA12 and NEA23 respectively. Regarding NEA12, of the 882 metabolites present in vitro, 56% (496) were also found in planta. A greater proportion of NEA23-derived metabolites (257/336, 76%) were also found present in planta.

## 4. Discussion

Improved resistance to disease caused by phytopathogens in pasture grasses directly contributes to increases in pasture yield and seed production, as well as reduced mycotoxicosis in livestock and reduced risk of mycotoxin contamination in livestock products [16,19,42,45,68,69]. Definitive benefits to pastoral agriculture via endophyte-mediated disease resistance relies on effective protection of the host grass from phytopathogen infection via the production of bioprotective compounds. Endophyte-mediated disease resistance is an outcome of complex host-pathogen-endophyte interactions resulting in antimicrobial compound production, secretion, and distribution throughout the host plant. Thus, it is important to further understand endophyte-derived antimicrobials and their contribution to disease resistance of host grasses.

Different asexual *Epichloë* spp. strains, that varied in their host specificity and major known alkaloid profiles, were investigated for antifungal activity using an in vitro screening process. Strains were identified with strong in vitro bioactivity, and these were targeted for further investigation. SE (*Lp*TG-1) showed consistent broad-spectrum bioactivity against the pathogens tested. The novel strains NEA12 (*Lp*TG-3), NEA21, and NEA23 (*Fa*TG-3 strains) exhibited strong bioactivity against the phytopathogens tested and are of particular interest when considering other favourable attributes, such as insect control and animal safety, associated with the presence/absence of well characterized secondary metabolites [6,70]. These strains have great potential for successful utilization in pastural agriculture.

In this study in vitro dual culture assays were used to evoke a response from a phytopathogen in the presence of an endophyte. The response was observed as a change in phytopathogen growth area, growth density, and growth direction. Use of imaging software allowed for accurate and precise digital measurement of pathogen growth area followed by statistical comparison of results. Quantitative data analysis confirmed that bioactive strains NEA12, NEA21, NEA23, and SE exhibited differential antifungal activity. NEA21 and NEA23 significantly inhibited the growth of the grass pathogen *Ceratobasidium* sp., while NEA12 exhibited moderate inhibition. SE and NEA12 showed more promising activity against *Drechslera* sp. and *Fusarium* sp. compared to the other endophyte strains.

In addition to a reduction in phytopathogen growth, reduced hyphal density and growth of hyphae away from the endophyte provide further evidence that pathogen growth was inhibited when in proximity to the endophyte. Furthermore, in the presence of endophyte, *Drechslera* sp., exhibited a brownish-orange morphology, possibly because of a stress response from the pathogen [71,72].

The variation in bioactivity observed between strains highlights the importance of testing multiple endophyte strains, as well as phytopathogens, to determine the bioprotective potential of each endophyte. It may be the case that for effective disease control in the field more than one endophyte strain is required.

Microorganisms have evolved to produce secondary metabolites as an adaptation to serve physiological, social, and predatory functionalities that are not fulfilled by primary metabolism [73]. In many cases bioprotective compounds are unique to a species or strain [73,74,75,76]. In this study, the differential bioactivity observed between the strains examined indicates that there is variation in the production of bioactive metabolites and their composition; each strain produces a unique set of antifungal compounds. Variation in production of well characterized alkaloids within *Epichloë* spp. provides an example: NEA12, an *Lp*TG-3 endophyte strain produces epoxy-janthitrems [77]; NEA21 and NEA23, *Fa*TG-3 endophytes, produce lolines and peramine [63]; SE, an *Lp*TG-1 endophyte strain, produces peramine and the mammalian toxins ergovaline and lolitrem B [67,70]. 

There is some evidence that lolines, peramine, and ergot alkaloids do not exhibit antifungal activity [54]. Siegel and Latch examined antifungal bioactivity of *N*-acetyl loline, *N*-formyl loline, peramine, ergotamine tartrate, and ergonovine maleate using disk diffusion assays and did not observe bioactivity against the pathogenic fungi examined [54]. Thus, the metabolites responsible for antifungal activity of these endophyte strains are yet to be defined.

Antifungal activity may be due to several different mechanisms; production of fungal cell wall degrading enzymes, small molecule antifungal compounds and proteins, and by induced systemic resistance [74]. One way to differentiate these mechanisms is to examine extracts from fungi grown under conditions that preferentially yield small molecules and exclude any large peptides or proteins. To further investigate the antifungal activity of NEA12 and NEA23, mycelia were grown as liquid cultures and the mycelial pellet and media supernatant were extracted in 80% aqueous methanol. Use of methanol allows for extraction of a variety of small molecules within a larger polarity range (polar to partially non-polar). The methanol will also cause precipitation of large peptides and proteins, reducing the number of potentially biologically active molecules to be investigated. Liquid cultures provide larger mycelial yields in a shorter period of time, as well as allow for separation of mycelia and media [55,63], thus facilitating analysis of bioactivity of the secretome and intracellular metabolome separately. Use of liquid cultures also allows for isolation and characterization of bioactive compounds once bioactivity is confirmed [54,78].

Agar-well diffusion assay results confirmed the stability of bioactivity in NEA12 and NEA23 using different culture conditions. Comparison between the secretome (MS) and the intracellular metabolome (MP) shows that NEA12 and NEA23 impede pathogenic growth by secreting antifungal metabolites into the culture media. This outcome also indicates that production of secreted antifungal compounds is constitutive, although it would not be surprising if the presence of the pathogen further stimulated production of antifungals as secondary metabolite production by endophytic fungi can be triggered by environmental pressures [1,79]. The inhibitory activity displayed by NEA12 MS and NEA23 MS are consistent with the dual culture assay results, where NEA23 exhibited stronger bioactivity against *Ceratobasidium* sp. compared to NEA12; and NEA12 inhibited the growth of *Fusarium* sp. while NEA23 did not. The consistent nature of endophyte bioactivity suggests that the suite of bioactive compounds produced under two different culture conditions are both the same and constitutively expressed.

Importantly, the in vitro antifungal phenotypes observed were consistent; observed in independent isolates, across duplicate assays and under two different culture environment conditions. Thus, while beyond the scope of this study, the bioactive compounds should be discoverable using bioassay guided fractionation and isolation [70,80,81].

It is also important to note here that previously isolated and characterized antifungal proteins or volatile antifungal metabolites from *Epichloë* spp. are not likely to be responsible for the bioactivity observed in this study. In contrast to other studies the extraction method and freeze drying used in this study targets the isolation of non-volatile small molecules which are likely be of high to medium polarity [5,47,51,53].

To be of use in bioprotection, bioactive compounds must be produced in sufficient quantities in planta, and also be distributed to where they are required. To investigate this aspect further, LCMS/MS/MS analysis was performed on fungal culture extracts from the endophyte secretome (NEA12 MS and NEA23 MS) as well as extracts of leaf material from the corresponding symbiota (NEA12 PE and NEA23 PE). In the first instance, presence of the major alkaloids produced by *Epichloë s*pp. endophytes was confirmed [1,2,3,4,5]. Presence of peramine in NEA23 and epoxy janthitrem I in NEA12 was confirmed both in vitro and in planta.

Venn diagrams summarising the metabolic features produced by the two endophytic strains showed that NEA12 and NEA23 produced 268 and 29 unique compounds, present both in vitro and in planta, respectively. Considering that the bioactive compounds of interest are stable and constitutively expressed, metabolites that are uniquely present in NEA12 in vitro and in planta, or NEA23 in vitro and in planta, are the best candidates for further investigating the unique/differential biological activity of these strains. While some metabolites are unique, a large portion of predicted endophyte-derived metabolites (228) are also found in common between the two endophyte strains. It is also possible that a combination of both unique and common metabolites is acting synergistically to produce the observed antifungal activity. Future studies are required to isolate and characterize antifungal metabolites and determine their role in disease resistance in planta.

This study provides methods for assessing the bioactivity of isolates of asexual *Epichloë* spp. endophytes and shows that the strains investigated here constitutively secrete stable antifungal metabolites under different culture conditions. Thus, the compounds responsible for the bioprotection phenotype observed in each strain should be discoverable using bioassay guided fractionation and isolation. Another important outcome of this study is that the endophyte strains examined exhibit differential antifungal activity, this phenotypic variation highlights the importance of evaluating the bioactive properties of multiple endophyte strains for inhibition of more than one phytopathogen. The presence of metabolites, identified by LCMS analysis, that are both unique and common to strains in vitro and in planta provides candidates for further investigation. Discovery of the endophyte-derived compounds responsible for disease resistance will enable selection of strains that enhance pasture production and produce animal safe feed by reducing mycotoxins.

## 5. Concluding Remarks

Identification of *Epichloë* spp. endophyte-derived antimicrobial compounds and determining their bioprotective properties is key to understanding the complex process of endophyte-mediated disease resistance in *Lolium* spp. This study focused on utilizing in vitro assays to identify and characterize the bioprotective properties of selected endophyte strains. The work presented here provides a solid basis enabling future studies focusing on isolation and characterization of individual compounds using the methods described here to confirm bioprotective properties in vitro. Once the bioactive metabolites are well characterized the presence and distribution in planta can be defined*,* a necessary step to determine the utility of the bioprotective agents against fungal diseases in the plant-endophyte symbiota. Ultimately, knowledge regarding bioactivity of individual endophyte strains can be used to improve disease resistance of *Lolium*-based pastures for the betterment of pasture production and animal welfare.

## Figures and Tables

**Table 1:** Selected *Lolium* spp. associated *Epichloë* spp. endophytes.

Endophyte Taxon	Examples of Strains	Native Host
*Epichloë festucae* var. *lolii* (*Lp*TG-1)	SE, AR1, NEA2, NEA3, NEA6, Endo5	perennial ryegrass
*Lp*TG-2	NEA4, NEA11, AR6	perennial ryegrass
*Lp*TG-3	AR37, NEA12	perennial ryegrass
*Epichloë coenophiala* (*Fa*TG-1)	E34, AR542	tall fescue
*Fa*TG-3	NEA21, NEA23	tall fescue

*Lp*TG—*Lolium perenne* Taxonomic Group; *Fa*TG—*Festuca arundinaceum* Taxonomic Group.

**Table 2:** Disease threat to perennial ryegrass, their causative agents and observed production loss.

Disease/Common Name	Causative Organism	Effect on Pasture/Seed Production	References
Crown rust	*Puccinia coronata*	Yield reduction 30–40%	[16,17,18]
Grey leaf spot	*Pyricularia grisea*	Up to 90% pasture loss	[19,20,21,22]
Brown blightand net blotch	*Drechslera* sp.	Dry matter and herbage yield reduction	[15,23]
Stem end rust	*Puccinia graminis*	Seed yield reduction, dry matter reduction	[24,25]
Blind seed disease	*Gloeotinia temulenta*	Seed yield reduction, reduced seed germination 50–90%	[26,27,28]
Snow mold	*Microdochium nivalae*	Seedling damage leading to reduced yield, seed loss	[15,29,30]
Yellow patch	*Ceratobasidium cereale*	Reduced yield	[31,32]
Damping off	*Fusarium* sp.	Reduced yield and dry matter	[33,34,35]
Bacterial wilt	*Xanthomonas transluscens*	Reduced yield 20–40%	[14,36,37,38]
Ryegrass mosaic virus	RgMV	Reduced dry matter yield loss 21–30%	[39,40]

**Table 3:** Summary of pathogens selected for this study.

Species	Accession Number	Disease	Host/Source
*Ceratobasidium* sp.	VPRI 22537	yellow patch/sharp eye spot	*Triticum aestivum* (wheat)
*Drechslera* sp.	VPRI 12962	leaf spot	*Briza maxima* (rattle grass)
*Fusarium* sp.	VPRI 43403	*Fusarium* patch	*Lolium perenne* (perennial ryegrass)
*Colletotrichum graminicola*	VPRI 32315	anthracnose	*Triticum aestivum* (wheat)

**Table 4:** *Epichloë* spp. strains isolated from perennial ryegrass (*Lolium perenne*).

*Epichloë* spp. Strain	Endophyte Taxon	Known Major Alkaloids	References
NEA21	*Fa*TG-3	lolines, peramine	[6]
NEA23	*Fa*TG-3	lolines, peramine	[6]
NEA12	*Lp*TG-3	epoxy-janthitrems	[55]
SE	*Lp*TG-1	lolitrem B, ergovaline, peramine	[6,55]

**Table 5:** Mycelia yield and media supernatant yield (after freeze drying) from 1 L PDB culture of NEA12 and NEA23.

Strains	Mycelia Yield	Media Supernatant Yield after Freeze Drying
NEA23	36.75 g	13.32 g
NEA12	13.21 g	10.11 g

## Supplementary Materials

The following are available online at https://www.mdpi.com/2076-2607/8/6/955/s1. **Table S1:** Antifungal activity of *Epichloë* spp. endophytes against selected grass pathogens. **Figure S1:** Dual culture assay for growth inhibition of pathogens *Ceratobasidium* sp. by four *Epichloë* spp. strains (in rows) NEA12 (*n =* 5), NEA23 (*n =* 5), NEA21 (*n =* 5), positive control SE (*n =* 5), and negative control (pathogen alone) (*n =* 5). From left to right (in columns) it shows the growth of pathogen from day 2–8. The images are of a typical representative of the five replicates. **Figure S2:** Dual culture assay for growth inhibition of pathogens *Drechslera* sp. by four *Epichloë* spp. strains (in rows) NEA12 (*n =* 5), NEA23 (*n =* 5), NEA21 (*n =* 5), positive control SE (*n =* 5), and negative control (pathogen alone) (*n =* 5). From left to right (in columns) it shows the growth of pathogen from day 2–8. The images are of a typical representative of the five replicates. **Figure S3:** Dual culture assay for growth inhibition of pathogens *Fusarium* sp. by four *Epichloë* spp. strains (in rows) NEA12 (*n =* 5), NEA23 (*n =* 5), NEA21 (*n =* 5), positive control SE (*n =* 5) and negative control (pathogen alone) (*n =* 5). From left to right (in columns) it shows the growth of pathogen from day 2–8. The images are of a typical representative of the five replicates. **Figure S4**: Dual culture assay for growth inhibition of pathogens *Ceratobasidium* sp., *Fusarium* sp*.,* and *Drechslera* sp. by four *Epichloë* spp. strains (in rows) NEA12 (*n =* 3), NEA23 (*n =* 3), NEA21 (*n =* 3), SE (*n =* 3), and negative control (pathogen alone) (*n =* 3). **Figure S5:** Agar-well diffusion assay for pathogens *Ceratobasidium* sp. in the presence of (from left to right): NEA23 MS (*n* = 5); NEA23 MP (*n* = 5); NEA12 MS (*n* = 5); NEA12 MP (*n* = 5); antifungal compound carbendazim (1 mg/mL) (*n* = 5); 80% methanol (*n* = 5); and sterile water (*n* = 5). From top to bottom (in rows) it shows the growth of pathogen from day 3–6. The images are of a typical representative of the five replicates. **Figure S6:** Agar-well diffusion assay for pathogens *Fusarium* sp. in the presence of (from left to right): NEA23 MS (*n* = 5); NEA23 MP (*n* = 5); NEA12 MS (*n* = 5); NEA12 MP (*n* = 5); antifungal compound carbendazim (1 mg/mL) (*n* = 5); 80% methanol (*n* = 5); and sterile water (*n* = 5). From top to bottom (in rows) it shows the growth of pathogen from day 3–8. The images are of a typical representative of the five replicates. **Figure S7:** Agar-well diffusion assay for pathogens *Ceratobasidium* sp. (top panel) and *Fusarium* sp. (bottom panel) in the presence of (from left to right): NEA23 MS (*n* = 5); NEA23 MP (*n* = 5); NEA12 MS (*n* = 5); NEA12 MP (*n* = 5); antifungal compound carbendazim (1 mg/mL) (*n* = 5); 80% methanol (*n* = 5); and water (*n* = 5). The images are of a typical representative of the five replicates. **Figure S8:** LCMS chromatograms of a) NEA12 in planta extract and b) NEA12 fungal culture extract showing the presence of epoxy janthitrem I (C_39_H_52_O_7_N *m*/*z* = 646.3752 Δ 2.5 ppm) eluting at 11.14 (±0.02 min). **Figure S9:** LCMS chromatograms of a) 80% methanol extract of perennial ryegrass plants with NEA23 b) 80% methanol extract of 2-week-old NEA23 PDB culture showing the presence of peramine (C_12_H_18_ON_5_ *m*/*z* = 248.1509) eluting at 3.52 (±0.02 min).
