# Supplementary material for: Novel Antifungal Activity of Lolium-Associated Epichloë Endophytes
Source: Microorganisms. 2020 Jun 24;8(6):955. doi: 10.3390/microorganisms8060955 (PMC7355949; doi:10.3390/microorganisms8060955)
Supplement: Supplementary file 1 [file microorganisms-08-00955-s001.pdf]

Supplementary material

Table S1: Antifungal activity of *Epichloë* spp. endophytes against selected grass pathogens.

Endophyte strain		Pathogen		
Strain ID	Taxon	*Colletotrichum graminicola*	*Ceratobasidium cereale*	*Drechslera brizae*
440364	*Fa*TG-1	Strong	Strong	Strong
AR542	*Fa*TG-1	Moderate	Strong	Strong
E34	*Fa*TG-1	Weak	Strong	Weak
NEA13	*Fa*TG-1	Moderate	Strong	Strong
NEA14	*Fa*TG-1	Moderate	Strong	Moderate
NEA15	*Fa*TG-1	Moderate	Strong	Strong
NEA16	*Fa*TG-1	Moderate	Strong	Strong
NEA22	*Fa*TG-1	Strong	Moderate	Moderate
NEA27	*Fa*TG-1	Weak	Weak	Moderate
NEA30	*Fa*TG-1	Moderate	Strong	Strong
NEA17	*Fa*TG-2	Moderate	Strong	Strong
NEA34	*Fa*TG-2	Moderate	Strong	Strong
610918	*Fa*TG-3	Moderate	Strong	Strong
AR510	*Fa*TG-3	Weak	Strong	Not tested
NEA21	*Fa*TG-3	Moderate	Moderate	Strong
NEA23	*Fa*TG-3	Moderate	Strong	Strong
NEA33	*Fa*TG-4	Moderate	Moderate	Weak
NEA18	*Fa*TG-5	Moderate	Strong	Strong
NEA19	*Fa*TG-5	Moderate	Strong	Strong
15285	*Lp*TG-1	Not tested	Not tested	Weak
AR1	*Lp*TG-1	Weak	Moderate	Weak
NEA10	*Lp*TG-1	Weak	Weak	Weak
NEA3	*Lp*TG-1	Weak	Weak	Weak
RU03001-60_E09	*Lp*TG-1	Weak	Strong	Moderate
SE	*Lp*TG-1	Weak	Strong	Strong
18-C1	*Lp*TG-2	Weak	Strong	Not tested
NEA11	*Lp*TG-2	Weak	Moderate	Not tested
FR15598_15310	*Lp*TG-3	Strong	Strong	Moderate
NEA12	*Lp*TG-3	Strong	Strong	Moderate
E1	*Lp*TG-4	Weak	Moderate	Weak

Strong
Moderate
Weak
Not tested

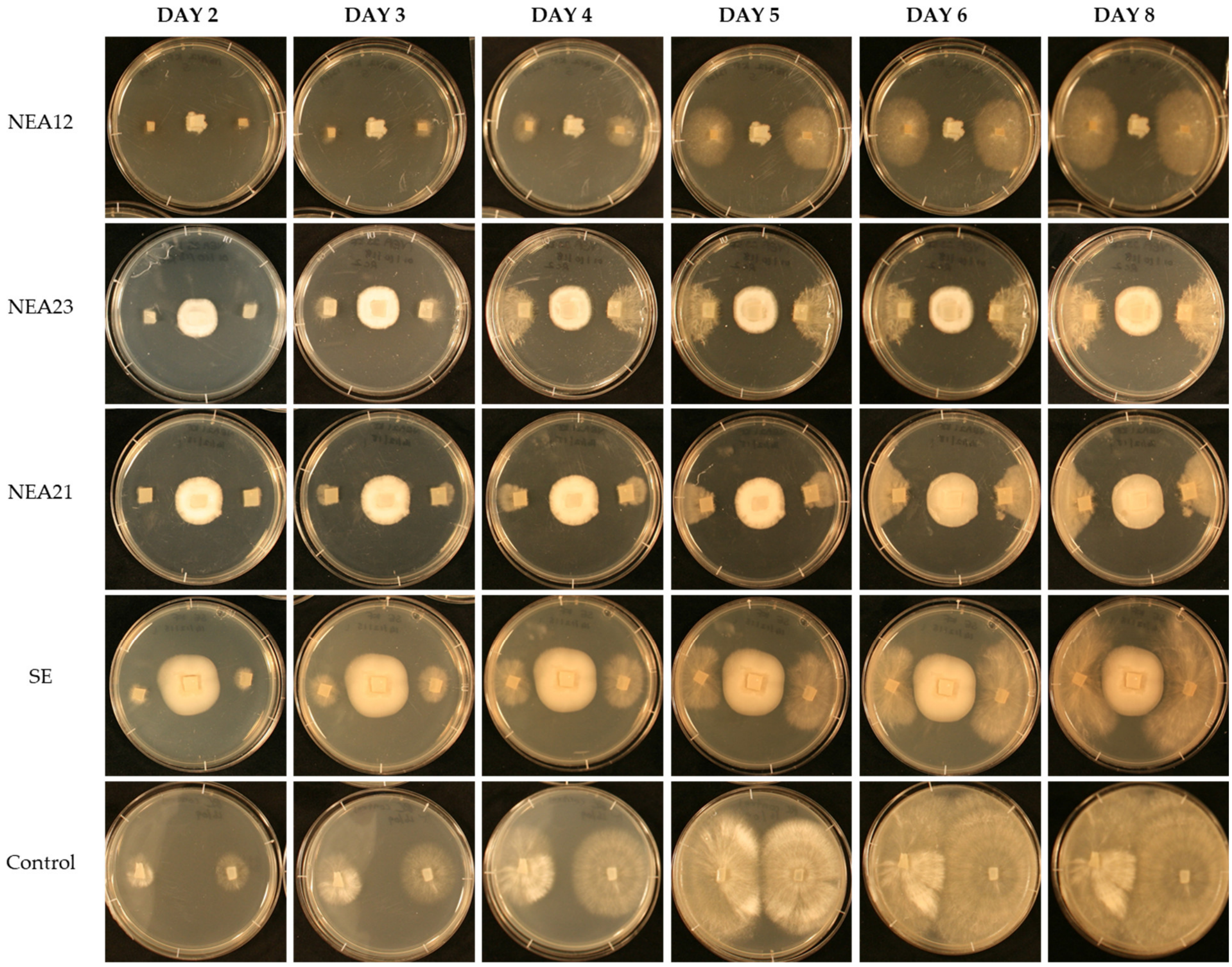

Figure S1: Dual culture assay for growth inhibition of pathogens *Ceratobasidium* sp. by four *Epichloë* spp. strains (in rows) NEA12 (*n*=5), NEA23 (*n*=5), NEA21 (*n*=5), positive control SE (*n*=5) and negative control (pathogen alone) (*n*=5). From left to right (in columns) it shows the growth of pathogen from day 2-8. The images are of a typical representative of the 5 replicates.

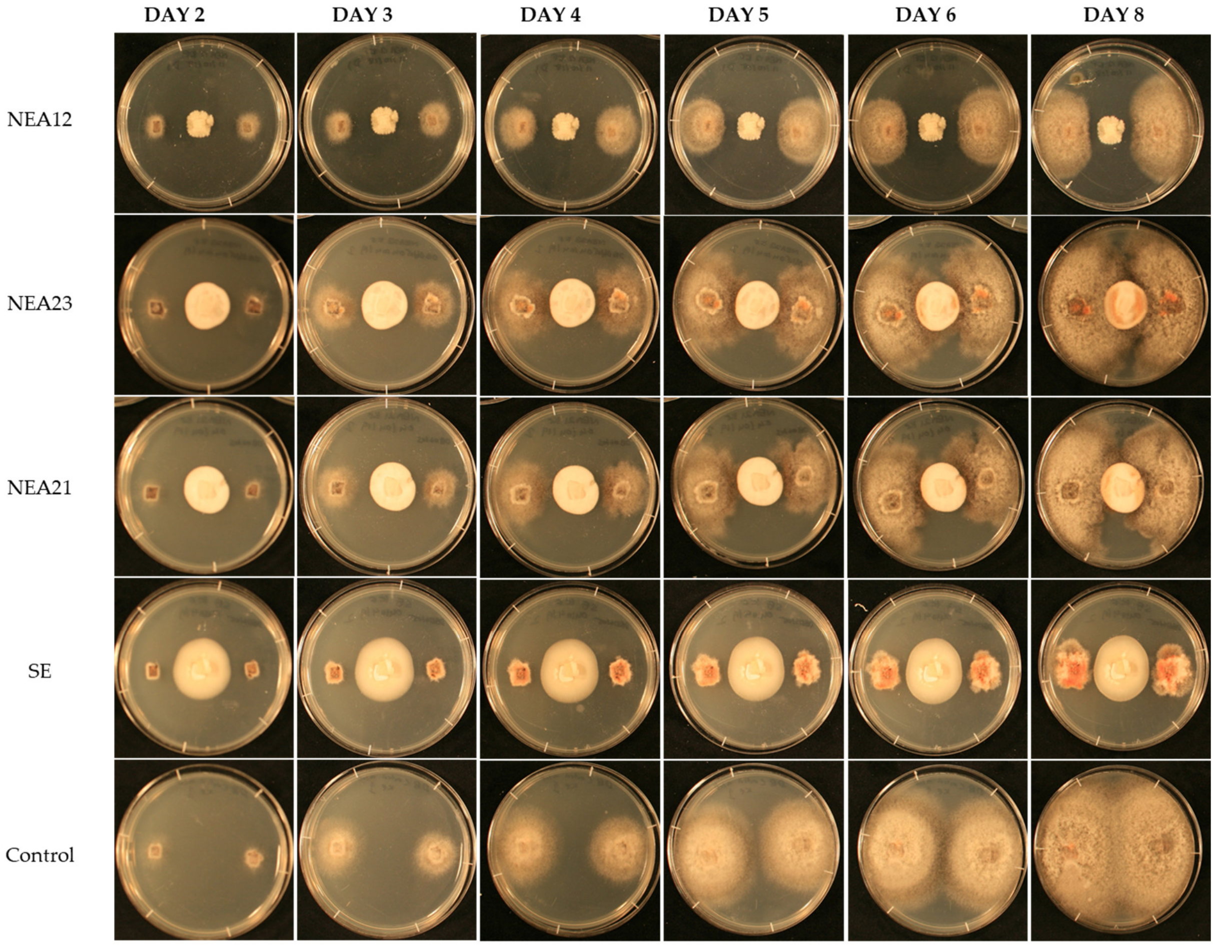

Figure S2: Dual culture assay for growth inhibition of pathogens *Drechslera* sp. by four *Epichloë* spp. strains (in rows) NEA12 (*n*=5), NEA23 (*n*=5), NEA21 (*n*=5), positive control SE (*n*=5) and negative control (pathogen alone) (*n*=5). From left to right (in columns) it shows the growth of pathogen from day 2-8. The images are of a typical representative of the 5 replicates.

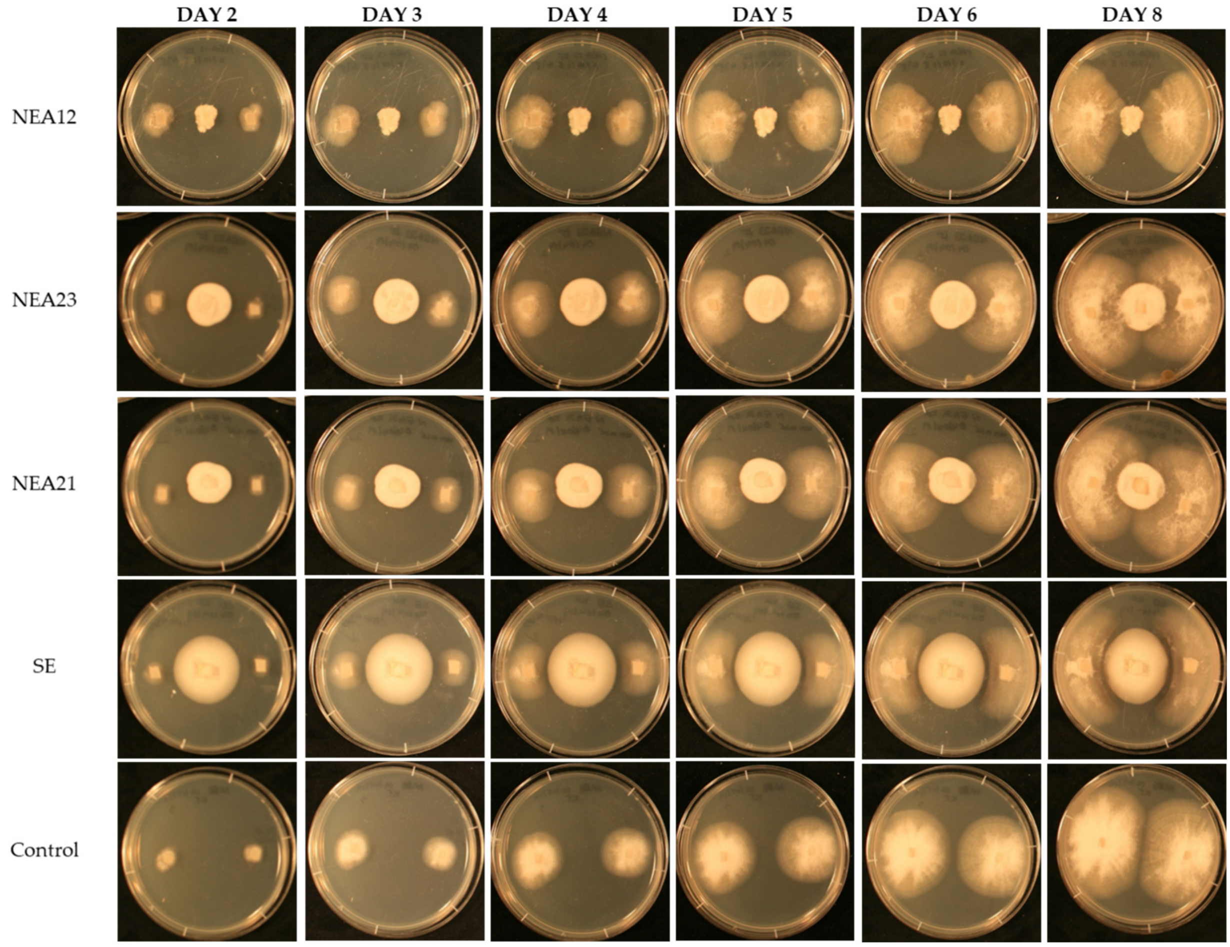

Figure S3: Dual culture assay for growth inhibition of pathogens *Fusarium* sp. by four *Epichloë* spp. strains (in rows) NEA12 (*n*=5), NEA23 (*n*=5), NEA21 (*n*=5), positive control SE (*n*=5) and negative control (pathogen alone) (*n*=5). From left to right (in columns) it shows the growth of pathogen from day 2-8. The images are of a typical representative of the 5 replicates.

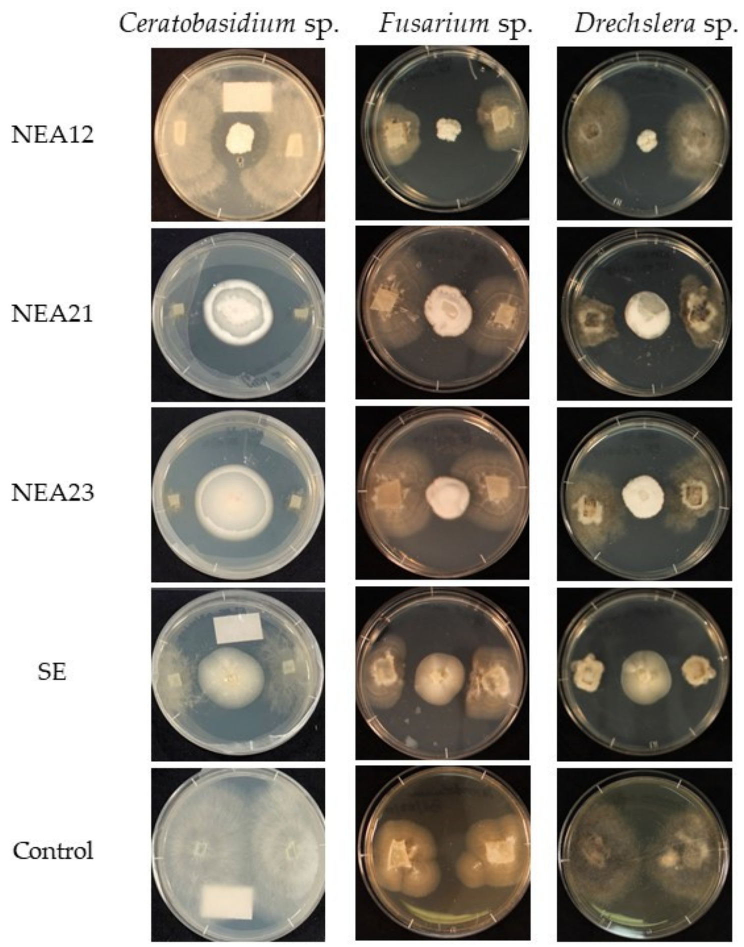

Figure S4: Dual culture assay for growth inhibition of pathogens *Ceratobasidium* sp., *Fusarium* sp. and *Drechslera* sp. by four *Epichloë* spp. strains (in rows) NEA12 (*n*=3), NEA23 (*n*=3), NEA21 (*n*=3), SE (*n*=3) and negative control (pathogen alone) (*n*=3).

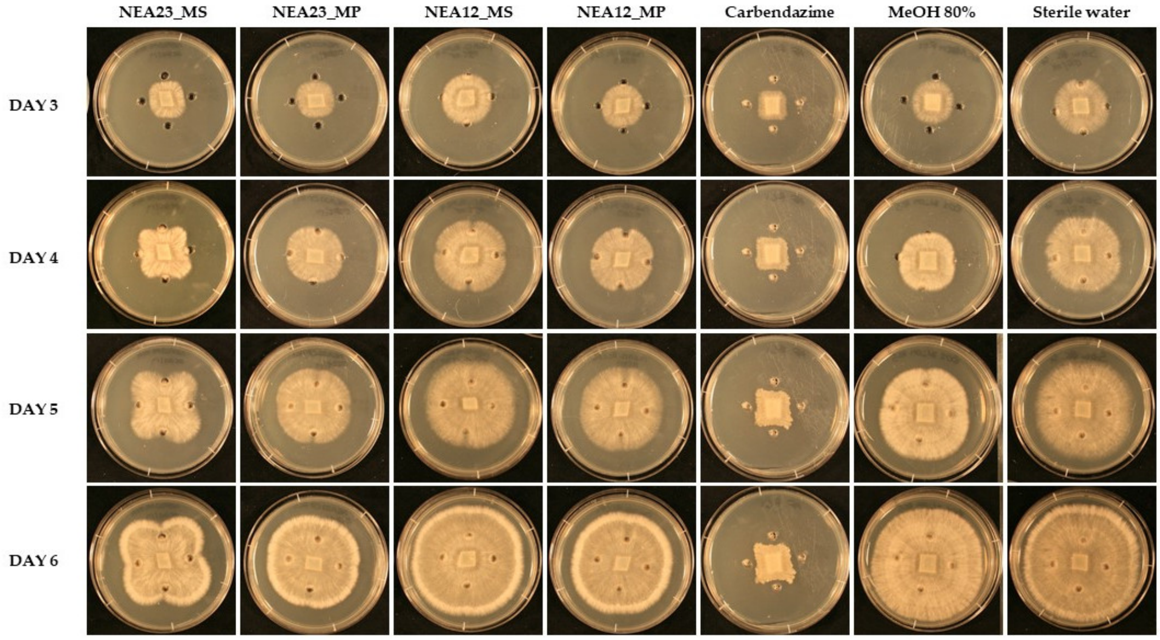

Figure S5: Agar-well diffusion assay for pathogens *Ceratobasidium* sp. in the presence of (from left to right): NEA23 MS (*n*=5); NEA23 MP (*n*=5); NEA12 MS (*n*=5); NEA12 MP (*n*=5); antifungal compound carbendazim (1 mg/ml) (*n*=5); 80% methanol (*n*=5); and sterile water (*n*=5). From top to bottom (in rows) it shows the growth of pathogen from day 3-6. The images are of a typical representative of the 5 replicates.

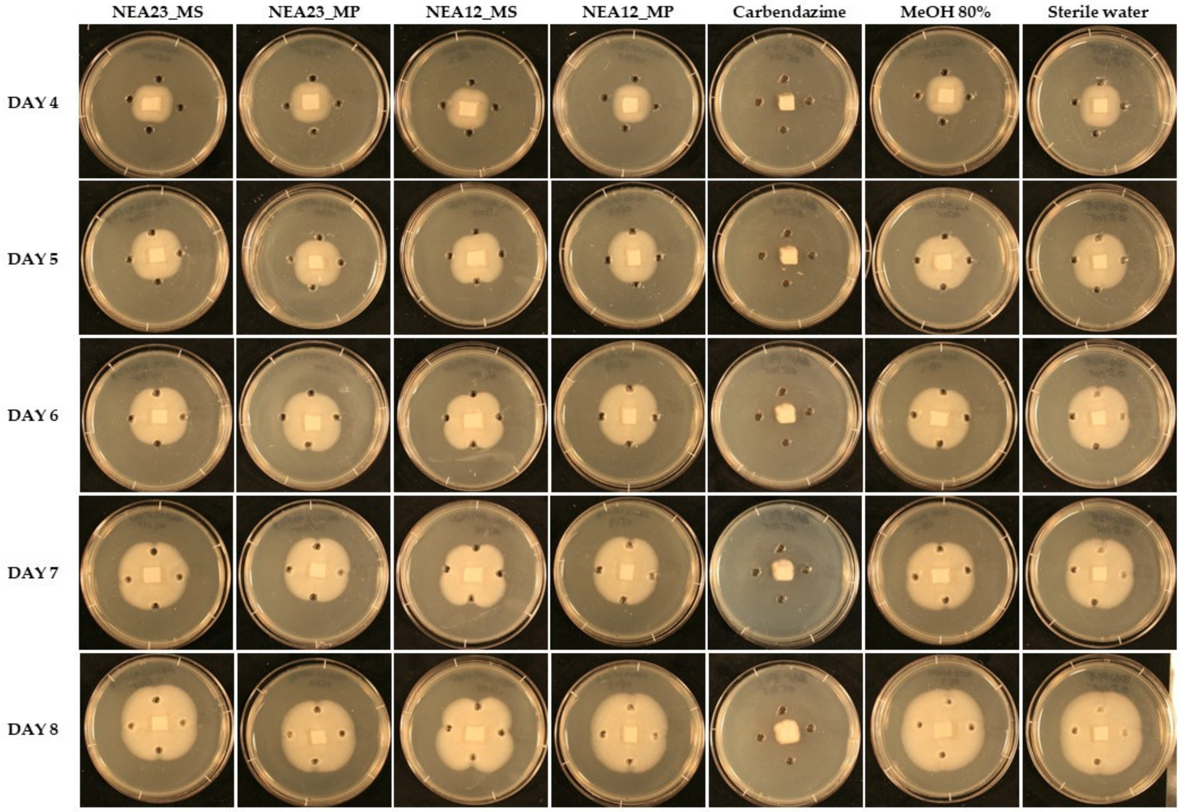

Figure S6: Agar-well diffusion assay for pathogens *Fusarium* sp. in the presence of (from left to right): NEA23 MS (*n*=5); NEA23 MP (*n*=5); NEA12 MS (*n*=5); NEA12 MP (*n*=5); antifungal compound carbendazim (1 mg/ml) (*n*=5); 80% methanol (*n*=5); and sterile water (*n*=5). From top to bottom (in rows) it shows the growth of pathogen from day 3-8. The images are of a typical representative of the 5 replicates.

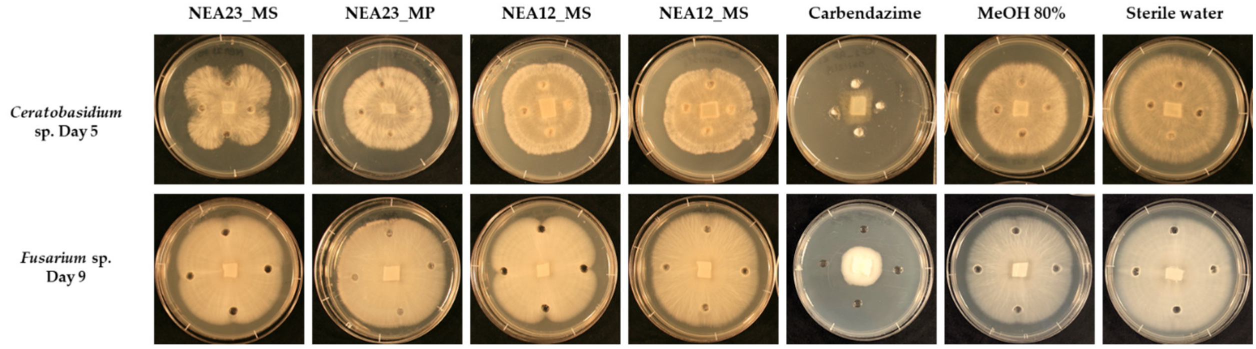

Figure S7: Agar-well diffusion assay for pathogens *Ceratobasidium* sp. (top panel) and *Fusarium* sp. (bottom panel) in the presence of (from left to right): NEA23 MS (*n*=5); NEA23 MP (*n*=5); NEA12 MS (*n*=5); NEA12 MP (*n*=5); antifungal compound carbendazim (1 mg/ml) (*n*=5); 80% methanol (*n*=5); and water (*n*=5). The images are of a typical representative of the 5 replicates.

a)

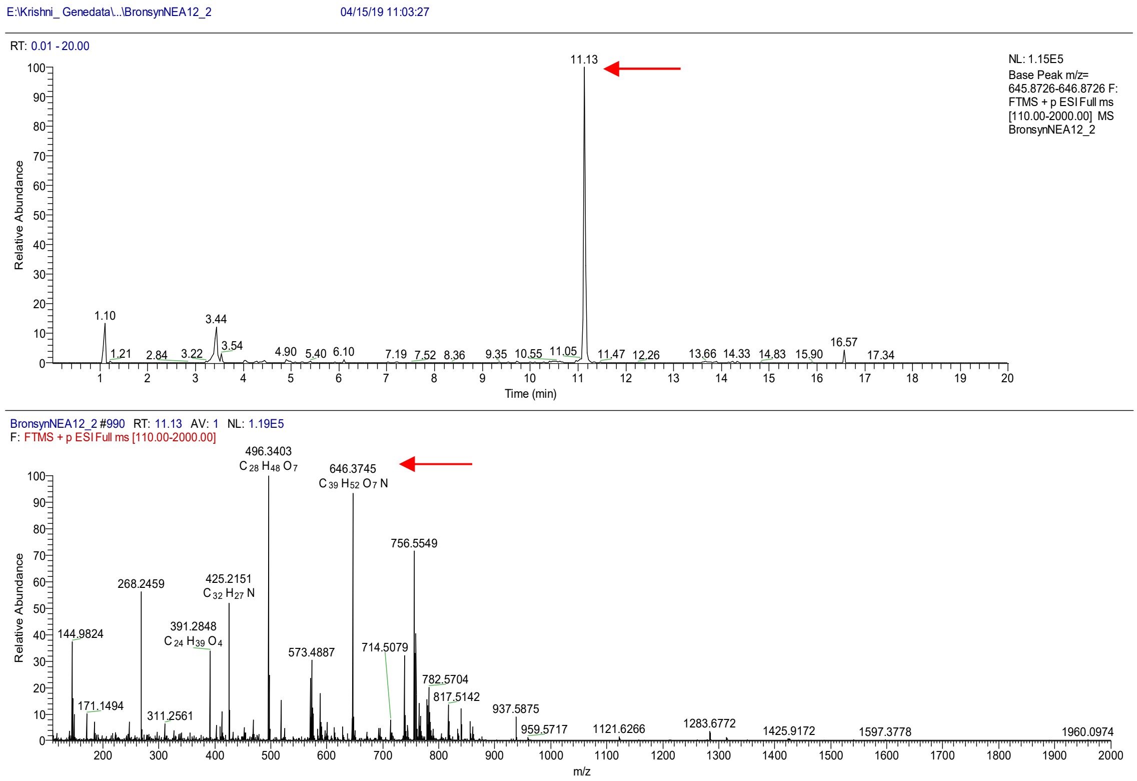

b)

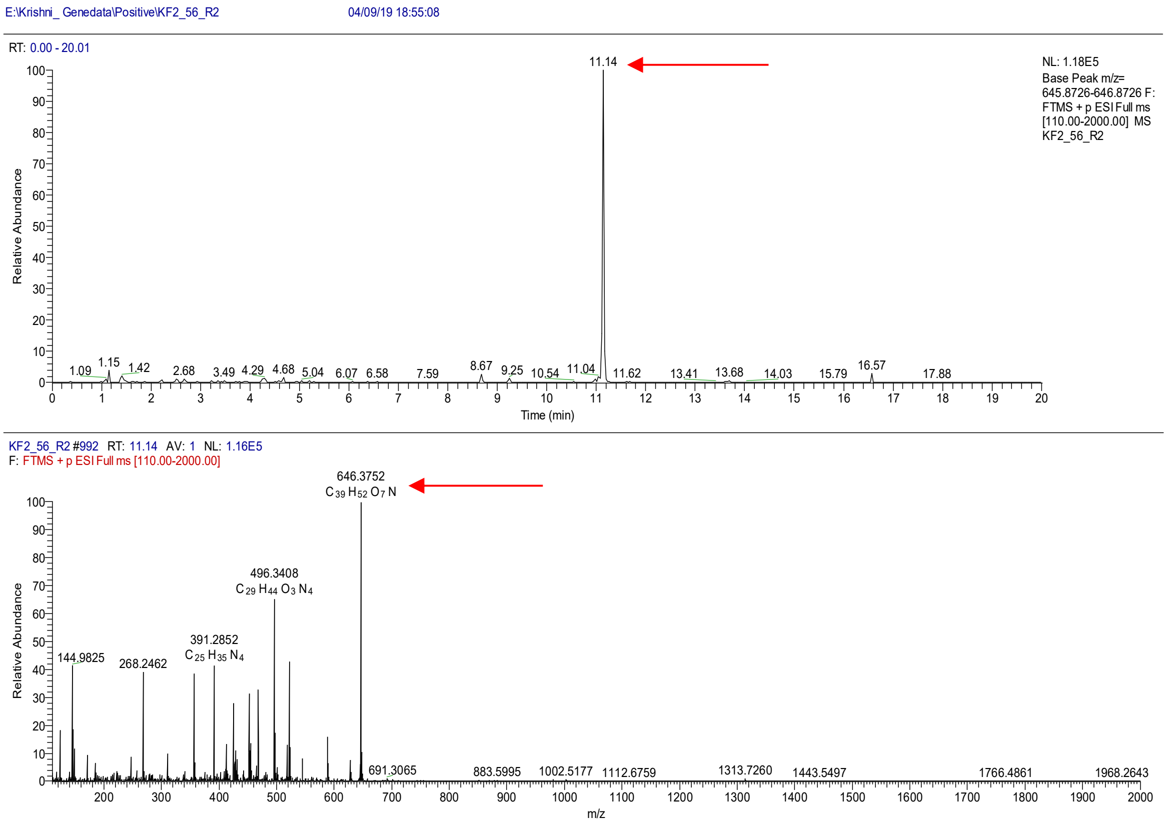

Figure S8: LCMS chromatograms of a) NEA12 in planta extract and b) NEA12 fungal culture extract showing the presence of epoxy janthitrem I ($C_{39}H_{52}O_7N$ *m/z* = 646.3752 Δ 2.5 ppm) eluting at 11.14 (±0.02 min) [1].

a)

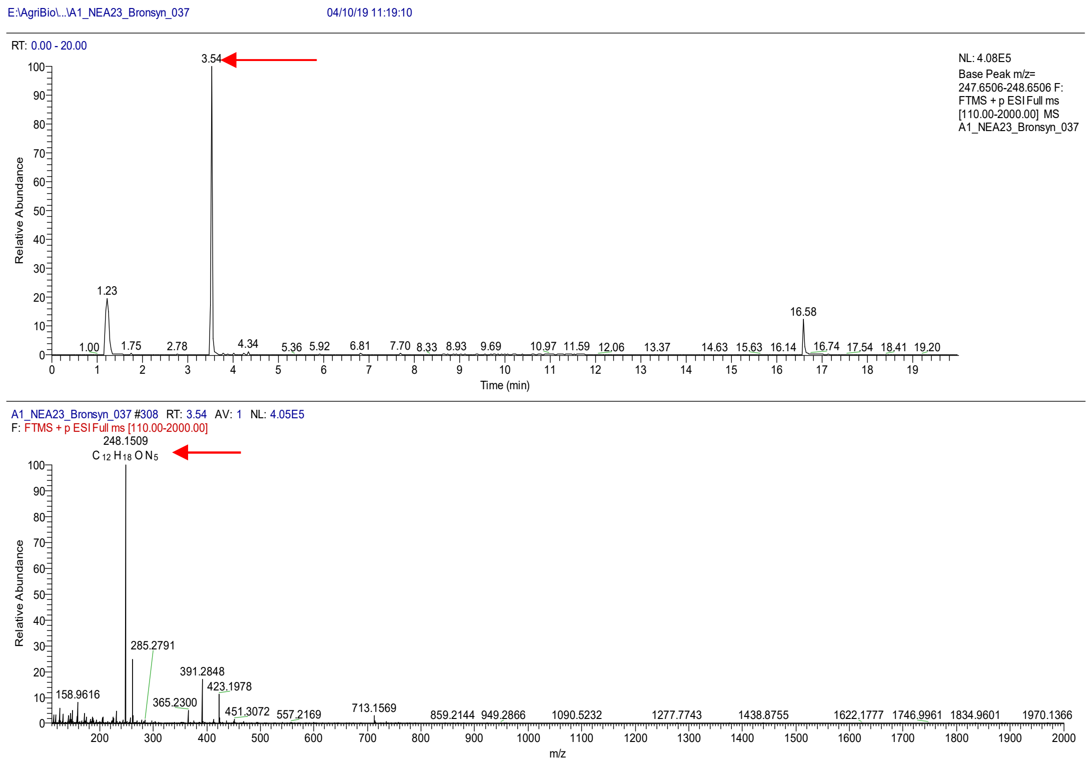

b)

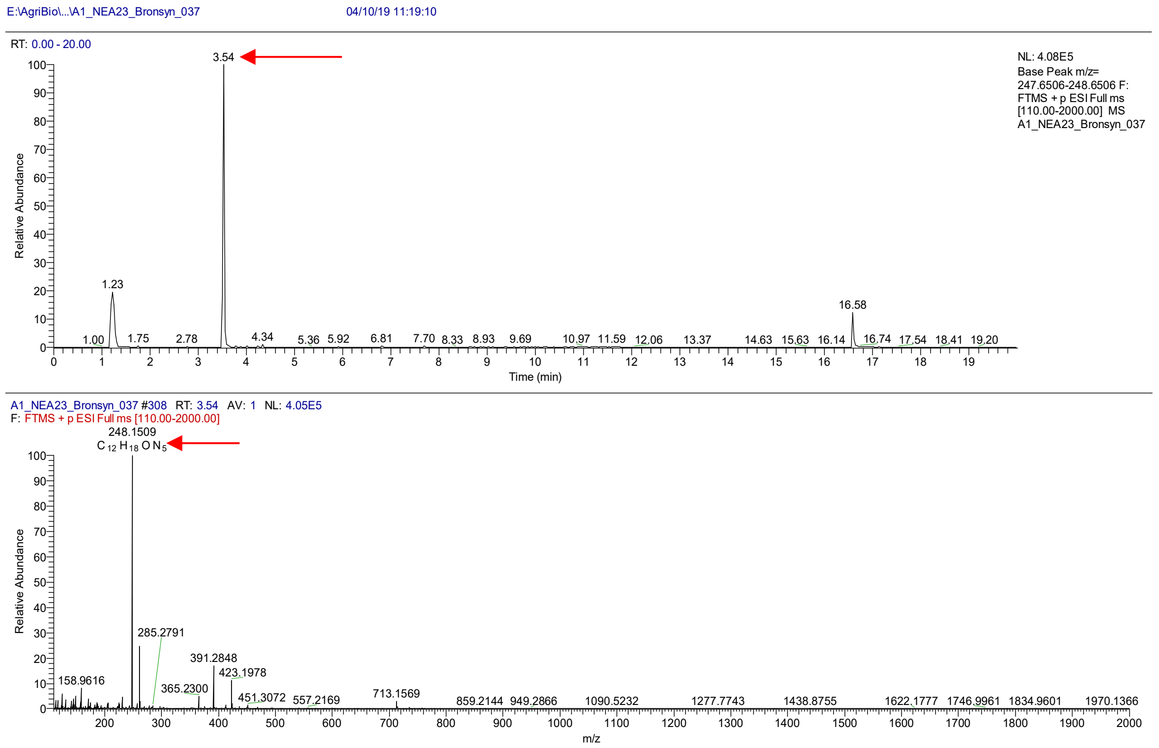

Figure S9: LCMS chromatograms of a) 80% methanol extract of perennial ryegrass plants with NEA23 b) 80% methanol extract of 2-week-old NEA23 PDB culture showing the presence of peramine ($C_{12}H_{18}ON_5$ – *m/z* = 248.1509) eluting at 3.52 (±0.02 min) [2].

1. Ludlow, E.J.; Vassiliadis, S.; Ekanayake, P.N.; Hettiarachchige, I.K.; Reddy, P.; Sawbridge, T.I.; Rochfort, S.J.; Spangenberg, G.C.; Guthridge, K.M. Analysis of the Indole Diterpene Gene Cluster for Biosynthesis of the Epoxy-Janthitrems in Epichloë Endophytes. *Microorganisms* **2019**, *7*, 560.

2. Reddy, P.; Deseo, M.A.; Ezernieks, V.; Guthridge, K.; Spangenberg, G.; Rochfort, S. Toxic indole diterpenes from endophyte-infected perennial ryegrass *Lolium perenne* L.: isolation and stability. *Toxins (Basel)* **2019**, *11*, 16.
